# Geospatial and Time Trend of Prevalence and Characteristics of Zero-Dose Children in Nigeria from 2003 to 2018

**DOI:** 10.3390/vaccines10091556

**Published:** 2022-09-18

**Authors:** Ryoko Sato

**Affiliations:** Center for Health Decision Science, Department of Global Health and Population, Harvard T.H. Chan School of Public Health, Boston, MA 02120, USA; rsato@hsph.harvard.edu

**Keywords:** zero-dose children, Blinder–Oaxaca decomposition analysis, Nigeria

## Abstract

Introduction: While recent years have observed a substantial improvement in vaccination coverage among children in developing countries, many children are still left out and remain unvaccinated. This study analyzes the trend of the prevalence and characteristics of zero-dose children in Nigeria over time. Methods: Using data from the Demographic and Health Survey in Nigeria from 2003 to 2018, I analyzed the prevalence and determinants of zero-dose children who had not received any DTP vaccine by geographical zone and over time. In addition, I conducted Blinder–Oaxaca decomposition analysis to evaluate the reasons for the change in the prevalence of zero-dose children over time. Results: The overall prevalence of zero-dose children reduced from over 60% in 2003 to 40% in 2018 in Nigeria. Rural areas had a higher prevalence of zero-dose children than urban areas and the gap was consistent over time. Southern zones consistently had a lower prevalence of zero-dose children, but northern zones observed more reductions in the prevalence of zero-dose children. The mother’s education and wealth level in a household are strongly associated with a lower likelihood of having zero-dose children. In both urban and rural areas, an improvement in the mother’s education level strongly explained the reduction in zero-dose children over time, while an increase in the wealth level also explained the reduction in zero-dose children in rural areas. Conclusions: While Nigeria has observed a substantial reduction in the prevalence of zero-dose children in the 15 years since 2003, the pattern of and explanatory factors for the reduction differ by geographical region. This analysis can be useful for identifying a targeting strategy to further reduce the prevalence of zero-dose children in Nigeria in the future.

## 1. Introduction

Vaccination is one of the most effective and cost-effective ways to mitigate health burdens from vaccine-preventable diseases in low- and middle-income countries. It saves 3.5 to 5 million lives every year [1]. The global coverage of basic vaccines (DTP3) reached over 80% among children in 2019 [2].

However, over 17 million children worldwide still have not received any basic vaccines [3], half of which live in just 12 countries, including Nigeria [4]. Indeed, Nigeria is one of the five countries that account for two-thirds of all zero-dose children in the world, and it contributes to 30% of the total number of unvaccinated children under five [5].

In Nigeria, vaccination coverage has been limited. The vaccination coverage was merely 36% for measles and 21% for DPT3, and 27% of children had never received any vaccination in 2003. However, the country has observed a substantial improvement since then. By 2018, the national vaccination coverage against measles reached 54% and DTP3 coverage reached 50%. The proportion of children who never received any vaccination was reduced to 19% [6]. However, the coverage is still limited.

The determinants of vaccination have been extensively investigated in the literature. In the existing literature reviews, the sociodemographic variables of mothers and households, such as the mother’s education and the wealth level of households, are often referred to as strong determinants of vaccination behaviors for children [7,8].

While the determinants of vaccination have been extensively studied in the past, studies that have investigated factors explaining the improvement in the level of vaccination over time have been extremely limited, although it is of importance for the policy implication of how we can improve vaccine uptake over time.

Thus, this paper studies the geographical differences in the progress of vaccination coverage, especially in terms of zero-dose children, and examines factors that have contributed to the reduction in zero-dose children over time in Nigeria.

## 2. Methods

### 2.1. Data

The dataset used in the analysis was the Nigeria Demographic and Health Survey (DHS) conducted between 2003 and 2018. The data are publicly available. The sample for the DHS in each wave was representative at national and state levels. The data contained information on children’s vaccination status, including whether each dose of vaccine was received by children aged between 0 to 36 months old within the 5 years prior to the survey.

### 2.2. Analysis

This paper first estimated the prevalence of zero-dose children over time by geographical regions in Nigeria. Then, using ordinary least-squares (OLS) regression, I estimated the correlation between various sociodemographic characteristics and the likelihood of a child being zero-dose. It is important to note, however, that the analysis was correlational, but not causal, and the results should be interpreted as such.

Finally, I conducted Blinder–Oaxaca decomposition analysis to evaluate factors that explained the change in the prevalence of zero-dose children over time in Nigeria. Blinder–Oaxaca decomposition is a statistical tool that enables us to explore how differences in an outcome variable across different groups or over time can be decomposed into explained and unexplained parts [9]. Within the explained and unexplained parts, decomposition analysis allows us to investigate which covariates attribute to the changes in the outcome variables and their extent. Details of Blinder–Oaxaca decomposition on the conceptual framework as well as on the software package used for this analysis can be found in the paper by Jann [9].

### 2.3. Outcome Variable

The main outcome variable is whether a child is considered as a zero-dose child or not. From the DHS data, vaccination records of the respondents’ children were used to define zero-dose children. Following the definition from GAVI [2], a child was defined as a zero-dose child if s/he did not receive the first dose of the DPT or pentavalent vaccine (DPT1/Penta1).

### 2.4. Covariates

I used various sociodemographic characteristics to evaluate if they were correlated with the likelihood of a child being zero-dose and if the change in these variables over time explained the change in the prevalence of zero-dose children over time.

The covariates included in the analysis were the mother’s age, child’s age, mother’s education level, household’s wealth level, and the place of residence (rural/urban). This information all originated from the Nigeria DHS.

## 3. Results

**Figure 1:**
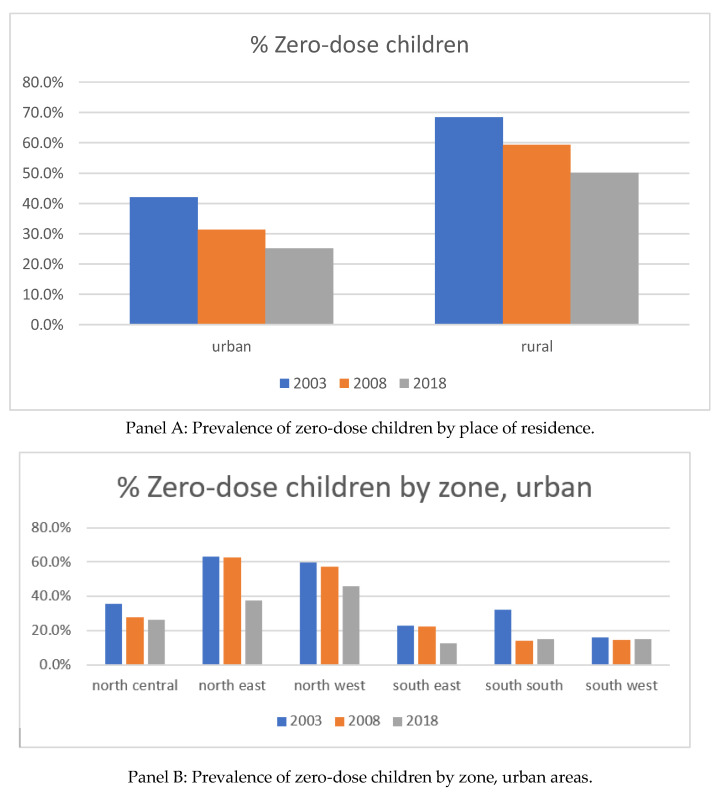

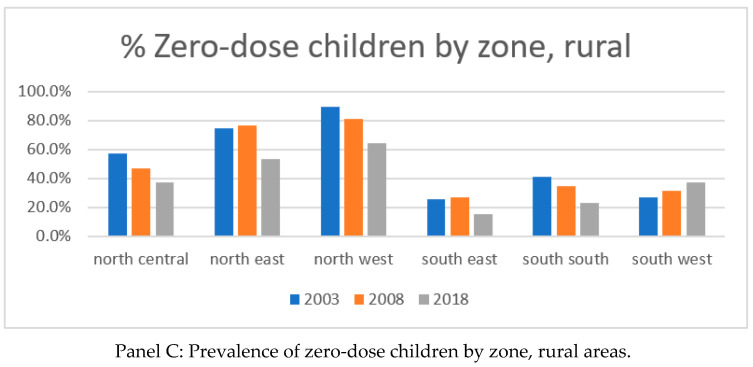
Prevalence of zero-dose children over time.

Figure 1 Panel A presents the prevalence of zero-dose children over time in Nigeria. The prevalence of zero-dose children was much higher in rural areas than in urban areas in 2003: 68.4% vs. 42.0%, respectively. Although both rural and urban areas experienced a substantial reduction in the prevalence of zero-dose children over time, the gap in the prevalence of zero-dose children by area did not narrow down, with 50.1% occurring in rural areas and 25.2% in urban areas in 2018.

Figure 1 Panels B and C present the prevalence of zero-dose children by zone in urban and rural areas, respectively. In urban areas, the prevalence of zero-dose children decreased in all zones. Some zones, including the north-east, south-east, and south-south, observed a substantial reduction in prevalence from 2003 to 2018 of over 40%. In the rural area, the prevalence mostly decreased over time, except in the south-west zone, which experienced a constant increase from 2003 to 2018 of 27.0% to 37.4%. The urban area in the south-west zone also experienced the smallest decrease in prevalence among all zones. The south-east and south-south zones observed the largest reduction (40.8 and 43.8% reduction, respectively) in the prevalence in rural areas, which was consistent with the observations in the urban area.

Table 1 presents the determinants of zero-dose children in each year. The trend was overall consistent in any year. Older age of mothers, higher education level of mothers, and higher household wealth level were all significantly and negatively correlated with the prevalence of zero-dose children in all years.

**Figure 2:**
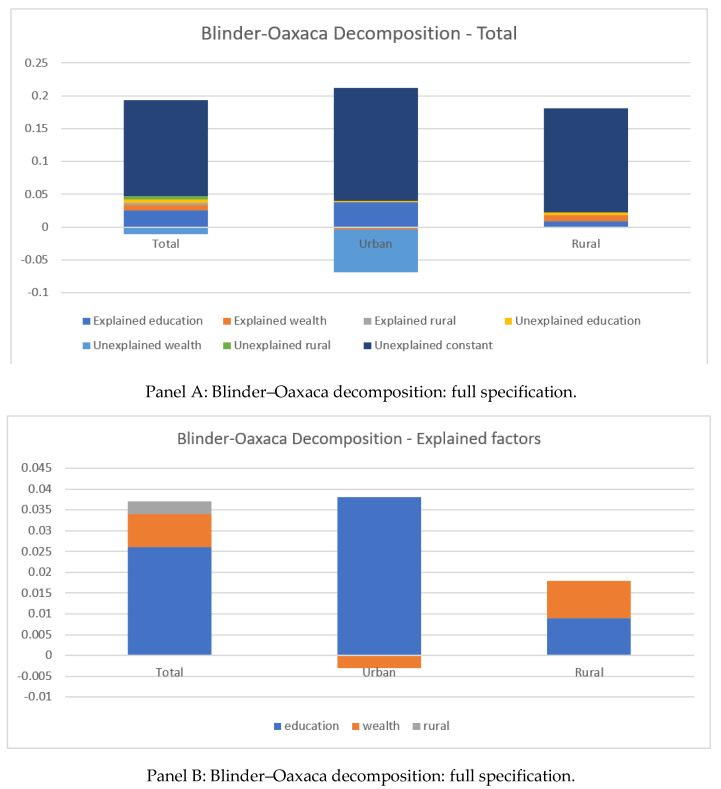
Blinder–Oaxaca decomposition for the change in the prevalence of zero-dose children over time.

Figure 2 presents the result of the Blinder–Oaxaca decomposition by the area of residence (urban/rural). Figure 2 Panel A shows that the change in the proportion of zero-dose children over time was largely due to unexplained factors. Among the explained factors, the reduction in the prevalence of zero-dose children in the urban area was primarily attributed to the improvement in the mothers’ education levels, while the improvement in household’s wealth and mothers’ education were equally important in explaining the reduction in the prevalence of zero-dose children in the rural area (Panel B).

**Figure 3:**
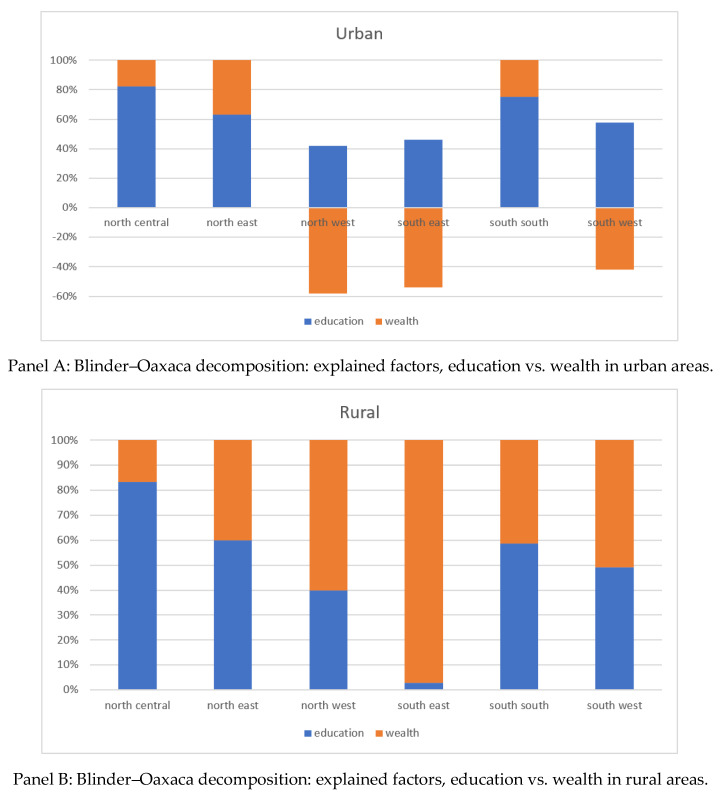
Blinder–Oaxaca decomposition for the change in the prevalence of zero-dose children over time.

Figure 3 presents the result of Blinder–Oaxaca decomposition in each zone. In urban areas, the improvement in education attainment among mothers was a dominant factor that explained the improvement in all zones (Panel A). On the other hand, the improvement in the wealth level also explained a substantial portion of the reduction in the prevalence of zero-dose children in some zones, especially in the north-central, north-east, and south-south zones.

In rural areas, on the other hand, the reduction in the prevalence of zero-dose children was attributed to the improvement in both education and wealth levels in all zones (Panel B). However, the relative importance of the improvement in education level to that of wealth level varied by the zone. For example, the improvement in education contributed to the reduction in zero-dose children in the north-central zone by 78.9%, followed by the north-east zone (60.0%) and south-south zone (58.7%). Meanwhile, the contribution of the improvement in wealth level was rather dominant in the south-east zone (91.7%), north-west zone (56.3%), and south-west zone (50.9%).

## 4. Discussion

This paper reports the prevalence of zero-dose children over time, as well as evaluates the factors that have contributed to the reduction in the prevalence of zero-dose children in Nigeria. Three waves of the nationally representative Demographic and Health Survey (DHS) between 2003 and 2018 were used for the analysis.

The baseline prevalence of zero-dose children was much higher in rural areas than in urban areas in 2003: 68% vs. 42%, respectively. In the 15 years from the base year, the prevalence has decreased substantially, with an approximately 18% decrease in both urban and rural areas. Rural areas always had a higher prevalence of zero-dose children than urban areas, and the gap was consistent and did not narrow over time. This result suggests that inequality in vaccination progress has remained the same between urban and rural areas over time.

The pattern of the reduction in the prevalence of zero-dose children varied by zone. Southern zones consistently had a lower prevalence of zero-dose children, but northern zones observed more reductions in the prevalence of zero-dose children over time.

The substantial reduction in zero-dose children was primarily due to unexplained factors. Among the explained factors, the improvement in education level among mothers was the dominant contributor to the reduction in the prevalence of zero-dose children. In rural areas, the improvement in wealth level also contributed substantially to the reduction in the prevalence of zero-dose children, but this was not the case for urban areas. This result was consistent with the determinants analysis, which revealed that mothers’ education and households’ wealth levels were negatively and significantly correlated with the low likelihood of having zero-dose children.

The results of this analysis on the determinants of zero-dose children were largely consistent with the recent analysis using data from sub-Saharan African countries [10,11].

Relevant to this study, Debie et al. [12] evaluated factors contributing to the improvement in the complete vaccination service utilization over time in Ethiopia and found that the improvement in vaccination coverage was attributed to socioeconomic characteristics, such as mothers’ education and households’ wealth levels. Although the analytical method used was different, my result was mostly consistent with their results.

This paper is the first to examine the factors contributing to the reduction in the prevalence of zero-dose children over time in sub-Saharan African countries. Depending on the area of residence (urban vs. rural), the relative importance of mothers’ education to households’ wealth levels was different: while education was important to reducing the prevalence of zero-dose children, regardless of whether they are in urban or rural areas, wealth was more important in rural areas than in urban areas. Substantial variations in the contributing factors by zone were also observed.

## 5. Conclusions

While Nigeria observed a substantial reduction in the prevalence of zero-dose children, the pattern of and explanatory factors for the reduction differed by geographical region. This result could be useful for developing a targeting strategy to further reduce the prevalence of zero-dose children in Nigeria in the future. Specifically, improving mothers’ knowledge might be efficient for reducing the prevalence of zero-dose children in urban areas, while taking a more holistic approach to improving the wellbeing of people, as well as educating mothers, can both be effective for mitigating the prevalence of zero-dose children in rural areas.

## Figures and Tables

**Table 1:** Determinants of zero-dose children.

	Zero-Dose Children			
	Total	Year = 2003	Year = 2008	Year = 2018
	(1)	(2)	(3)	(4)
Mother’s age	−0.005 ***	−0.008 ***	−0.005 ***	−0.003 ***
	(0.000)	(0.001)	(0.000)	(0.001)
Child’s age	−0.027 ***	−0.032 ***	−0.023 ***	−0.041 ***
	(0.002)	(0.004)	(0.002)	(0.004)
Primary education (comparison = no education)	−0.292 ***	−0.297 ***	−0.312 ***	−0.260 ***
	(0.005)	(0.015)	(0.007)	(0.010)
Secondary education (comparison = no education)	−0.442 ***	−0.437 ***	−0.478 ***	−0.396 ***
	(0.005)	(0.017)	(0.007)	(0.009)
Higher education (comparison = no education)	−0.490 ***	−0.457 ***	−0.531 ***	−0.451 ***
	(0.009)	(0.034)	(0.013)	(0.015)
Poorer (comparison = poorest)	0.007	0.035 *	0.012	−0.010
	(0.008)	(0.020)	(0.010)	(0.015)
Medium (comparison = poorest)	0.033 ***	−0.012	0.070 ***	0.002
	(0.007)	(0.022)	(0.010)	(0.013)
Richer (comparison = poorest)	−0.045 ***	−0.066 ***	−0.043 ***	−0.037 ***
	(0.006)	(0.017)	(0.008)	(0.011)
Richest (comparison = poorest)	−0.108 ***	−0.169 ***	−0.113 ***	−0.076 ***
	(0.007)	(0.021)	(0.009)	(0.013)
Rural (comparison = urban)	0.048 ***	0.048 ***	0.053 ***	0.043 ***
	(0.005)	(0.015)	(0.007)	(0.008)
Year = 2008 (comparison: year = 2003)	−0.068 ***			
	(0.007)			
Year = 2018 (comparison: year = 2003)	−0.166 ***			
	(0.007)			
_cons	0.981 ***	1.107 ***	0.926 ***	0.757 ***
	(0.012)	(0.033)	(0.015)	(0.020)
N	47,750	5165	25,239	17,346
r2	0.255	0.272	0.282	0.196

Notes: * Significant at the 90 percent confidence level. *** Significant at the 99 percent confidence level.

## Data Availability

Publicly available datasets were analyzed in this study. This data can be found here: https://dhsprogram.com/data/available-datasets.cfm.
